# Infective Spondylitis in Adults: A Journey Through Diagnosis, Management, and Future Directions

**DOI:** 10.3390/antibiotics14040391

**Published:** 2025-04-09

**Authors:** Woo-Seok Jung, Sung-Ryul Choi, Ji-Won Kwon, Kyung-Soo Suk, Hak-Sun Kim, Seong-Hwan Moon, Si-Young Park, Jae-Won Shin, Byung-Ho Lee

**Affiliations:** 1 Department of Orthopedic Surgery, Ewha Womans University Seoul Hospital, Ewha Womans University College of Medicine, Seoul 07804, Republic of Korea; 790359@ewha.ac.kr; 2 Department of Orthopedic Surgery, College of Medicine, International St. Mary’s Hospital, Catholic Kwandong University, Incheon 22711, Republic of Korea; srchoi1012@ish.ac.kr; 3 Department of Orthopedic Surgery, Spine and Spinal Cord Institute, Gangnam Severance Hospital, Yonsei University College of Medicine, Seoul 06273, Republic of Korea; kwonjjanng@yuhs.ac (J.-W.K.); sks111@yuhs.ac (K.-S.S.); 4 Department of Orthopedic Surgery, Yonsei University College of Medicine, Seoul 03722, Republic of Korea; haksunkim@yuhs.ac (H.-S.K.); shmoon@yuhs.ac (S.-H.M.); drspine90@yuhs.ac (S.-Y.P.); jaewuni@yuhs.ac (J.-W.S.)

**Keywords:** infective spondylitis, spinal infection, antibiotic therapy, biofilm, pyogenic spondylitis, epidural abscess

## Abstract

Infective spondylitis is a rare but potentially devastating spinal infection that requires timely diagnosis and appropriate treatment to prevent severe complications, including neurological deficits and spinal deformity. Despite advancements in diagnostic imaging, microbiological techniques, and antimicrobial therapies, clinical challenges persist because of the disease’s insidious onset, varied etiologies, and increasing antimicrobial resistance. This review article provides a comprehensive analysis of the current literature on the epidemiology, pathophysiology, diagnostic approaches, and treatment strategies for infective spondylitis.

## 1. Introduction

Since the 1990s, the incidence of spinal infections has risen because of factors such as an aging population, longer life expectancy among patients with chronic debilitating diseases, a growing number of immunocompromised individuals and intravenous (IV) drug receivers, and the increased application of spinal instrumentation surgeries, acupuncture, and epidural catheters for pain management. Further, the widespread availability of magnetic resonance imaging (MRI) has contributed to the increase in the definitive diagnoses of spinal infections. Achieving favorable outcomes in these cases depends on prompt diagnosis, followed by aggressive medical treatment and, when necessary, surgical intervention. The mortality rate of vertebral osteomyelitis is particularly high in patients with comorbidities, such as hemodialysis or impaired liver function, and the determination of prognostic factors and treatment strategies is crucial in these cases. This review investigates the epidemiology, pathophysiology, clinical features, investigations, and treatment of spondylodiscitis based on current evidence [1,2,3,4,5,6,7]. This review focuses exclusively on infective spondylitis in adults, as the pathophysiology, clinical presentation, and management can differ significantly from pediatric cases [8].

## 2. Vertebral Osteomyelitis

### 2.1. Overall

Vertebral osteomyelitis predominantly presents with nonspecific symptoms, which can delay diagnosis for several months. Spinal trauma, postsurgical complications, or hematogenous spread from a nearby site of infection may cause vertebral osteomyelitis. Without appropriate treatment, vertebral osteomyelitis poses a high risk of severe complications, including spinal deformity, paraplegia, and even death. Improving the clinical recognition of this condition is crucial for reducing the associated morbidity and mortality [2,5].

### 2.2. Epidemiology

The incidence of vertebral osteomyelitis differs depending on the region and population being investigated. The estimated incidence of vertebral osteomyelitis in the United States of America (USA) is 4.8 cases per 100,000 person-year and has been increasing over the last few decades. Vertebral osteomyelitis accounts for approximately 3–5% of all osteomyelitis cases annually in the USA [9,10]. Before the introduction of antibiotics, the mortality rate within 1 year was >25%, but currently, it is approximately 11% [9].

### 2.3. Etiology

A single pathogen infection typically causes vertebral osteomyelitis, with *Staphylococcus aureus* being the most prevalent, especially with the presence of hematogenous dissemination [11]. Therefore, if a patient has had *S. aureus* bacteremia within the last 3 months and demonstrates compatible findings on spine MRI, a vertebral osteomyelitis diagnosis can be presumed without requiring disc space aspiration or other tissue sampling. Conversely, coagulase-negative *staphylococci* and *Propionibacterium acnes* are predominantly implicated in exogenous osteomyelitis cases after spinal surgery, particularly with spinal fixation devices [5]. However, vertebral osteomyelitis caused by different pathogens may occur in endemic regions and immunocompromised individuals. Endemic pathogens include *Mycobacterium tuberculosis* and *Brucella* spp., whereas the *Mycobacterium avium* complex is frequently observed in patients with human immunodeficiency virus. Although rare, fungal vertebral osteomyelitis affects patients in endemic regions (e.g., histoplasmosis, blastomycosis, and Coccidioidomycosis) and those who are immunocompromised (*Aspergillus*), as well as IV drug receivers and individuals with indwelling IV catheters (*Candida* and *Aspergillus*) [5,11].

## 3. Discitis

### 3.1. Overall

**Figure 1:**
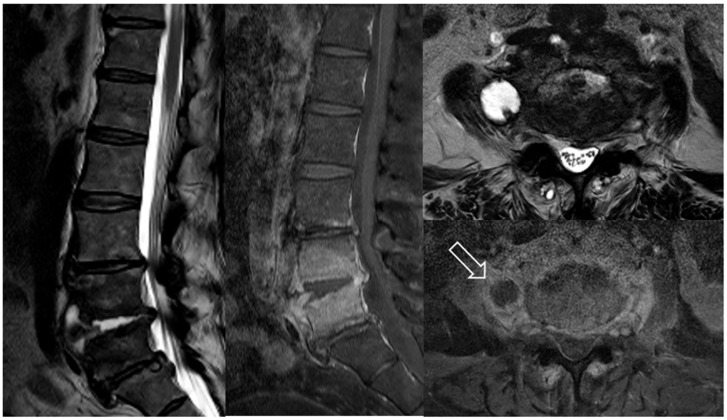
An 80-year old female patient presented to the emergency department with persistent lower back pain and fever. Magnetic resonance imaging(MRI) demonstrated signs consistent with spondylodiscitis at the L4–5 level, including disc space narrowing and endplate irregularity, as well as a rim-enhancing fluid collection in the right psoas muscle suggestive of an abscess. A white arrows indicate the location of the right psoas abscess on the axial image and the spondylodiscitis on the sagittal image.

Intervertebral discs are frequently referred to as the largest avascular structures of the human body. These conditions cause discitis to be an uncommon medical diagnosis. Discitis is relatively rare and is difficult to treat because of the minimal blood supply. Therefore, it often requires prolonged antibiotic treatment but may still leave behind an uncomplicated symptom resolution. Figure 1 illustrates vertebral osteomyelitis and discitis with psoas abscess [12,13].

### 3.2. Epidemiology

The incidence of discitis in the USA is approximately 0.4–2.4 per 100,000 person-year [12]. In general, discitis occurs more prevalently in pediatric patients compared to adults, probably related to the vascular supply of the intervertebral discs, which decreases with age [14]. However, a bimodal distribution of ages was observed, with another surge in incidence observed at approximately 50 years of age. Moreover, it exhibits a higher prevalence in males than in females [12]. In children, discitis is part of a spectrum of conditions including discitis, spinal osteomyelitis, and soft-tissue abscesses [14].

### 3.3. Etiology

Discitis may spread to the affected intervertebral disc via the bloodstream from a systemic infection such as a urinary tract infection (UTI). Various origins have been implicated, with UTIs, pneumonia, and soft-tissue infections being the most prevalent. No conclusive evidence has associated direct trauma with discitis. IV drug administration with contaminated needles provides direct access for various organisms to enter the bloodstream. In most cases, no other infection site is identified [15,16,17].

*S. aureus* is the most predominantly identified organism in discitis, although *Escherichia coli* and *Proteus* species are more prevalent in patients with UTIs. *Pseudomonas aeruginosa* and *Klebsiella* species are other Gram-negative bacteria observed in IV drug receivers, although less prevalent than *S. aureus*. Medical conditions that predispose patients to infections in other areas of the body, such as diabetes, acquired immunodeficiency syndrome, steroid use, cancer, and chronic renal insufficiency, are associated with discitis [18].

Disc space infection may also occur postoperatively, although it is relatively uncommon. Anterior cervical discectomy and fusion (ACDF) is associated with a low postoperative infection rate, typically ranging from 0.1% to 1.6% [19], while that of lumbar discectomy ranges between 0.2% and 1.2%, depending on patient factors and surgical conditions [12,14,18,20].

## 4. Epidural Abscess

### 4.1. Overall

**Figure 2:**
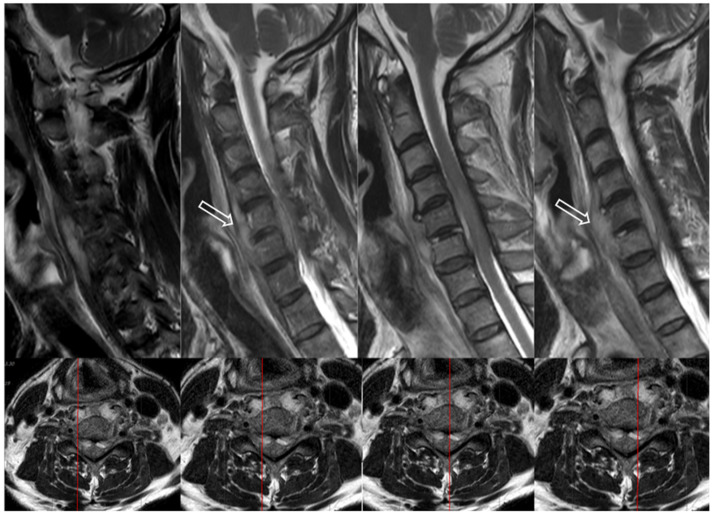
A 64-year-old male patient presented to the emergency room with quadriplegia. Magnetic resonance imaging(MRI) revealed an abscess within both longus coli muscles and discitis at the C5–6 level, along with an epidural abscess extending from C3 to C6, causing spinal cord compression. The white arrows indicate the abscess in the longus coli muscles.

Epidural abscess is a rare but significant purulent infection of the central and peripheral nervous system. Abscesses that are confined within the bony structures of the skull or spinal column can expand and compress the neurological structures, causing severe symptoms, permanent complications, or even death. An epidural abscess, unlike other spinal infections, directly compresses the nerve root and spinal cord, potentially causing neurological involvement such as sensory deficits or motor weakness. Early diagnosis and appropriate treatment prevent complications and cure many cases. Figure 2 illustrates a cervical epidural abscess with spinal cord compression [21,22,23,24,25,26].

### 4.2. Epidemiology

Overall, spinal epidural abscesses are rare. In particular, a retrospective study conducted from 2004 to 2014 at a large academic hospital in the USA reported an incidence of 5.1 cases per 10,000 admissions. A spinal epidural abscess is particularly rare in children, with less than one-third of affected children having an identifiable risk factor [27]. This incidence indicates an increase compared to previous decades. From 1990 to 2000, the incidence in Minnesota was 0.88 cases per 100,000 person-years [28]. Part of this increase may be related to improved diagnosis sensitivity and accuracy through MRI [27]. In the USA, the increasing incidence of spinal epidural abscesses may also be associated with the aging population and the growing number of patients undergoing invasive spinal procedures for anesthesia or pain control.

The median age at the onset of spinal epidural abscess is approximately 50 years, with the highest prevalence between ages 50 and 70 years, although it occurs at any age. Some studies have shown a higher incidence in male patients [21].

### 4.3. Etiology

Spinal epidural abscess develops as a secondary complication from any bacteremia-causing conditions [21]. Examples include injection drug administration, dental abscesses, infected catheters, and infective endocarditis, all of which are risk factors for spinal epidural abscesses.

Moreover, spinal procedures can cause spinal epidural abscesses, with epidural catheter placement being a significant risk factor. The incidence of spinal epidural abscess after epidural catheter placement ranges from 0.5% to 3% [29]. This risk is considerably lower when catheters are used for short durations, such as in obstetrical anesthesia [30]. Further, paraspinal glucocorticoid or analgesic injections cause spinal epidural abscess development [31]. Patients with vertebral osteomyelitis frequently develop spinal epidural abscesses. A study revealed that of 167 patients with hematogenous vertebral osteomyelitis, 64 (38%) had spinal epidural abscesses [32].

The primary bacterial pathogen responsible for spinal epidural abscess is *S. aureus*, accounting for approximately two-thirds of cases caused by pyogenic bacteria [33]. Among microorganisms isolated from the tips of epidural catheters, coagulase-negative staphylococci were the most commonly identified pathogens, accounting for approximately 41% of cases, followed by Staphylococcus aureus (35%) [34]. Other bacteria, such as Gram-negative *bacilli* and *streptococci*, can also cause the infection. Although rare, *M. tuberculosis* can also result in epidural abscesses [33,35].

## 5. Subdural or Intradural Abscess

Although extremely rare, subdural and/or intradural abscesses exist either as primary or secondary conditions and may require more emergent surgical decompression and antibiotic therapy [36,37,38].

Spinal subdural abscesses are extremely rare, with only a handful of cases reported in the literature. Clinical presentations typically include progressive back pain, neurological deficits, and evidence of subarachnoid block. Surgical findings have described subdural granulation tissue and encapsulated purulent collections, most commonly involving the thoracic or lumbar regions. Pathogens such as *Staphylococcus aureus* have been isolated. Although some patients recover with surgical drainage, neurological outcomes are often limited [39,40].

Intradural spinal abscesses are considerably less common than epidural abscesses, with most cases believed to result from hematogenous spread. Associations with prior epidural procedures or underlying spondylodiscitis have been reported but remain uncommon, possibly due to the filtering function of the epidural space. These abscesses typically affect older male patients, with a predilection for the lumbar spine. Despite their rarity, intradural abscesses represent a serious clinical concern. A high index of suspicion is warranted in patients presenting with intense back pain, progressive neurological deficits, and elevated inflammatory markers. Timely diagnosis is essential, as early surgical intervention combined with appropriate antibiotic therapy can significantly improve outcomes [36,41].

## 6. Paraspinal Muscle Abscess

### 6.1. Overall

To address the spread of infection into the paraspinal tissues typically, treatments of the underlying spinal infection are involved. Any adjacent anterior and lateral abscesses are usually drained concurrently when surgical debridement is performed for conditions such as spondylodiscitis. The iliopsoas abscess, which is the most prevalent paraspinal infection site, is classified into primary and secondary types. Primary iliopsoas abscess is rare and is typically caused by the hematogenous or lymphatic spread of a pathogen from a distant infection. In contrast, most cases are secondary iliopsoas abscesses, which usually originate from inflammatory conditions of the spine or skeleton [42].

### 6.2. Epidemiology

Paraspinal abscesses are rare medical emergencies, occurring in approximately 0.2–1.2 cases per 10,000 hospital admissions. A meta-analysis determined diabetes (15–53.7% of cases), spinal surgery (22%), and IV drug administration (8.8%) as significant risk factors for developing a paraspinal abscess. Further, some cases are associated with alcoholism and trauma. The source of infection in approximately one-third of cases remains unidentified. If untreated, the clinical outcome can be catastrophic [23].

### 6.3. Etiology

The most prevalent pathogens responsible are Gram-positive bacteria, particularly *S. aureus*. Gram-negative infections may also occur and are frequently related to ascending UTIs [43]. Tuberculosis (TB) should also be considered in the presence of epidemiologic risk factors. Less common pathogens include *Nocardia*, *Actinomyces* species, and fungi. Risk factors include previous back surgery, spinal surgery or instrumentation, back injury, and bacteremia. Patients receiving injection medications, immunosuppressed, and with diabetes were at a higher risk [42,43].

## 7. Diagnosis of the Spinal Infection

### 7.1. Symptoms

Spinal infections often present with nonspecific symptoms, which delays diagnosis. A systematic review [2] revealed that >90% of patients reported experiencing localized neck or back pain, making it the most prevalent symptom. However, only approximately half of the patients presented with a fever during diagnosis [44]. Further, the spinal range of motion is frequently restricted because of localized pain and muscle spasms [45,46]. Focal back pain, often exacerbated by percussion, has been reported in up to 90% of patients with spinal infections. Radicular symptoms and signs of spinal cord involvement have also been observed in up to 59% and 29% of cases, respectively, although the prevalence may vary depending on the underlying pathology and stage at diagnosis [47,48].

### 7.2. Laboratory Tests

The 2015 Clinical Practice Guidelines for the Diagnosis and Treatment of Native Vertebral Osteomyelitis in Adults, published by the Infectious Disease Society of America, recommend that clinicians consider the possibility of spinal infections in patients presenting with any of the following signs and symptoms [11]:

1. Worsening local pain accompanied by fever;
2. New or worsening local pain along with increased ESR or CRP levels;
3. New or worsening local pain with sepsis or infective endocarditis;
4. Fever combined with new neurological symptoms, with or without local pain;
5. New local pain after a recent episode of sepsis caused by *S. aureus.*

Although nonspecific, elevated ESR and CRP levels demonstrate high sensitivity—ranging from 94% to 100%—for detecting underlying spinal infections. These markers are particularly useful in evaluating patients with persistent back pain, helping to rule out infection or malignancy. Notably, white blood cell counts may remain within normal limits in up to 40% of patients with native vertebral osteomyelitis. Among these markers, CRP tends to normalize more quickly with appropriate treatment and may better reflect the patient’s clinical progress. However, these markers should be interpreted in the clinical context, as they may also be elevated in non-infectious inflammatory conditions [11,49].

Plasma procalcitonin has recently emerged as an important biomarker for distinguishing between bacterial infections and systemic inflammatory diseases. Patients with any bacterial infection typically have a procalcitonin level of >0.5 ng/mL (normal range: <0.05 ng/mL). Procalcitonin demonstrates variable sensitivity, which may be influenced by the presence of concurrent infections [50,51].

### 7.3. Imaging Tests

In the initial disease stages, plain radiographs can be obtained, although they are frequently normal, as characteristic features may not appear until after two to three weeks. Typical radiographic signs of spondylodiscitis involve the destruction of two adjacent vertebral bodies accompanied by the collapse of the intervening disc space. In rare cases, an infection that involves only a single vertebral body may present as a vertebral collapse comparable to a compression fracture [1].

MRI is considered the gold standard for diagnosing discitis, providing 92% specificity, 96% sensitivity, and 94% accuracy [52]. In acute cases, MRI typically demonstrates high fluid intensity. On T1-weighted images, the infection initially affected the anterolateral portion of the vertebral body, resulting in irregular signal intensity in the end plates, with edema spreading to the rest of the vertebral body and the adjacent disc. A pyogenic infection usually appears as a low signal on T1-weighted images and a high signal on T2-weighted images, with vertebral body contrast enhancements on T1 images after contrast administration.

CT scans are valuable in detecting paraspinal abscesses and bony sequestrate, as well as determining the best location and approach for a biopsy. Noteworthily, radiological results do not always align with the clinical presentation, as clinical symptoms may improve while radiological appearances are worsening. A reduction in contrast enhancement on an MRI can be an early sign of resolving infection; therefore, patients should be closely monitored with follow-up MRIs and careful clinical correlation during result interpretation [53,54].

The appearance of less common pathogens in spinal infections can include features, such as skip lesions, intraosseous abscesses, large paravertebral abscesses, and posterior element, spinal canal, and nerve root involvement, which indicate TB infection [55]. Brucella infection is characterized by an intact vertebral structure with signs of widespread intervertebral infection. The absence of T2-weighted enhancement after contrast administration is a notable feature of fungal infections [53].

A radioisotope bone scan with gallium is highly sensitive in detecting early changes in the disease process, demonstrating a 94% accuracy rate [12]. However, caution is required when interpreting gallium scans in leukopenic or elderly patients, as these individuals may experience relative ischemia of the end plate, which can influence the results [56].

Nuclear medicine imaging modalities, such as positron emission tomography—Computed tomography (CT) using fluorine-18 fluorodeoxyglucose and single-photon emission computed tomography/CT are also employed in diagnosing spondylodiscitis and are known to demonstrate higher sensitivity and specificity than bone scans [57].

### 7.4. Bacteriological Tests

To select the appropriate antibiotics, the causative organism needs to be identified. In patients with suspected spinal infections, it is recommended to culture specimens from the infection site and obtain two blood samples before starting antibiotic treatment [11]. For suspected cases of *Mycobacterium tuberculosis* spinal infection or in patients with a UTI history, sputum or urine cultures may also be required.

Specimens from the infection site are typically collected through percutaneous needle biopsy, guided by CT or fluoroscopy. The success rate of pathogen detection using this method varies between 41% and 90% [11,58]. The likelihood of detecting the pathogen is significantly higher in the presence of a psoas abscess, although specimens are not directly taken from the abscess [59]. If cultures are negative but a spinal infection remains suspected, a second biopsy is recommended. Detection rates were notably lower in patients who had received antibiotics before the biopsy. In such cases, a biopsy is advisable 1 or 2 weeks after stopping antibiotics, although detection rates may remain low [59]. An open biopsy is an alternative for patients with multiple negative biopsies, as it yields higher positive results compared to a percutaneous biopsy [11]. Overall, organisms are determined in 67–100% of spinal infection cases [58].

A PCR can be performed on spinal biopsy specimens and provides a rapid diagnostic tool for detecting M. tuberculosis. Although PCR may detect DNA from nonviable organisms, leading to false positives in patients who are undergoing or have completed treatment, during the initial diagnostic workup, a positive PCR is generally considered indicative of active infection. Compared to mycobacterial culture, which can take 6–8 weeks, PCR offers faster results with high sensitivity (around 88%) and specificity (approximately 95%), making it a valuable adjunct in early diagnosis [60,61,62].

## 8. Postoperative Infection

### 8.1. Overall

**Figure 3:**
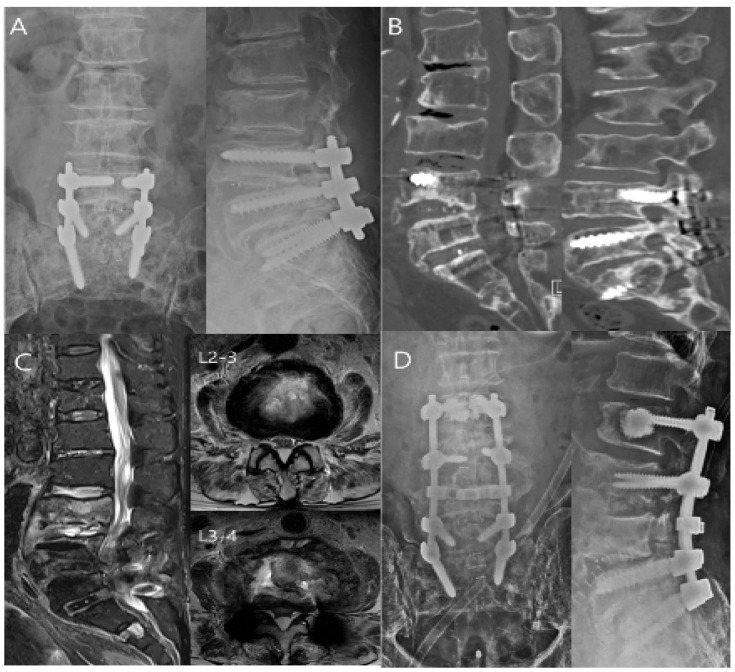
A 70-year-old woman with L4–L5–S1 spinal stenosis underwent partial laminectomy, posterior lumbar interbody fusion, and instrumentation. After four months, she returned with sudden severe back pain. The surgical site exhibited swelling and warmth, resulting in a suspected and confirmed late-postoperative infection via X-ray and CT scan (**A**,**B**). No evidence of implant loosening was observed. An initial incision and drainage surgery provided temporary relief, but signs of infection persisted, and the MRI revealed spondylodiscitis and worsening kyphotic deformity (**C**). She ultimately underwent revision surgery, including screw removal and extended fusion to L2 (**D**). The patient consented to publish her clinical images.

Postoperative spine infection can be a severe complication after spine surgery, affecting both short-term and long-term outcomes. Such infections significantly increase the risk of pseudoarthrosis, chronic pain, repeat surgeries, adverse neurological effects, poor long-term prognosis, and even death [63]. Figure 3 illustrates late postoperative infection [64].

### 8.2. Epidemiology

The incidence of infection after spine surgery varies widely, ranging from 0% to 18%, depending on the surgical type. Simple lumbar decompression or microdiscectomy exhibits a lower infection rate, approximately 0.6–3%, compared with instrumented fusion, which demonstrates an infection rate of approximately 6–18%. Further, the surgical approach influences the infection rate, with posterior surgery having a higher incidence of infection than anterior spinal surgeries [65].

### 8.3. Etiology

Several factors related to pathophysiology and microbiology cause postoperative spinal infections. The use of instrumentation in spinal surgery plays a crucial role in the development of these infections. Instrumentation causes local soft-tissue irritation, resulting in inflammation and seroma formation, thereby establishing an ideal environment for microorganisms to thrive. The adherence of bacteria to implant surfaces is facilitated by a polysaccharide biofilm called glycocalyx, which acts as a barrier against the host’s defense mechanisms and antibiotics [66]. Further, metallosis from the micromotion of the instrumentation causes granuloma formation, which provides another medium for bacterial colonization [66,67,68].

Various studies investigated the effect of preoperative epidural steroid injections (ESIs) on postoperative infection [69,70,71,72,73,74]. Kreitz et al. published results indicating an increased infection rate in patients who underwent fusion surgery and received ESI but not in decompression surgery. However, Lee et al. revealed that preoperative ESI is not a risk factor for postoperative infection [75,76]. ESI and acupuncture are frequently used as treatment methods for lumbar degenerative disease. Sung et al. reported that both acupuncture and ESIs performed more than 2 weeks before spinal surgery did not increase the risk of postoperative infection [7,77].

*S. aureus* remains the most commonly isolated organism in spinal SSIs. The prevalence of methicillin-resistant strains (MRSAs), however, varies depending on the clinical setting and geographic location [78,79,80]. Further, *Staphylococcus epidermidis* is increasingly predominant in postoperative infections. *E. coli* and *E. faecalis* are more prevalent in patients with incontinence or fecal contamination. Low-virulence microorganisms, such as *P. acnes*, can infect surgical wounds in patients with compromised immune systems. Late hardware infections, although generally rare, can involve Gram-negative rods and are more frequent in patients with trauma, those with severe neurological injuries, and immunocompromised individuals (with an injury severity score of >18). Patients with neuromuscular scoliosis, such as those with cerebral palsy or Duchenne’s muscular dystrophy, are at a higher risk due to poor bowel and bladder control, coupled with a lack of baseline mobility.

Polymicrobial infections are almost exclusively due to direct wound contamination during the postoperative period, frequently involving fecal or urinary contamination in patients with neuromuscular disorders [81,82].

## 9. Treatment of the Spinal Infection

Spinal infections typically require prolonged IV antibiotics or antifungal therapy, frequently resulting in extended hospital stays. Immobilization may be advised in cases of significant pain or potential spine instability. The initial treatment approach should be conservative, especially in early-stage cases with no or minor neurological deficits or when severe comorbidities limit surgical options [1,65,83,84]. Pyogenic vertebral osteomyelitis generally exhibits a favorable prognosis in the absence of structural instability and neurological deficits, considering that appropriate antibiotic therapy is administered [2,85]. However, in elderly patients with comorbidities, pyogenic spondylitis diagnosis is predominantly delayed, and concurrent infections in other organ systems are frequently observed [86]. These infections significantly affect clinical outcomes, potentially resulting in mortality despite appropriate treatment [85,87,88]. Clinically, negative initial nonoperative culture results are predominant. In such cases, spinal decompression and abscess drainage can be considered for patients with neurological deficits, progressive deterioration, or intolerable pain caused by an abscess, and the procedure can be performed using an endoscope [1,89,90,91]. Additionally, if mechanical instability is present before or after spinal decompression, early spinal instrumentation may be performed, considering the presence of a psoas abscess [92].

A broad range of antibiotics is available for the treatment of spinal infections. In critically ill patients or when cultures remain negative, empirical dual-agent therapy is typically recommended. This usually includes a combination of a third-generation cephalosporin or fluoroquinolone with an anti-Gram-positive agent such as clindamycin or vancomycin. Empirical regimens should be selected to cover both Gram-positive and Gram-negative organisms until the causative pathogen is identified, with options including clindamycin, vancomycin, or flucloxacillin combined with ceftriaxone, cefepime, or ciprofloxacin. Once culture results are available, antibiotics should be tailored accordingly. If *methicillin-sensitive Staphylococcus aureus* is isolated, anti-staphylococcal penicillins (e.g., nafcillin or flucloxacillin) or a first-generation cephalosporin is preferred. In cases involving methicillin-resistant strains, glycopeptides such as vancomycin or teicoplanin are recommended, with alternatives including linezolid or quinupristin-dalfopristin. For *Streptococcus* spp., penicillin G remains the first-line agent. When Gram-negative bacilli are implicated—particularly in infections originating from urinary tract sources—second- or third-generation cephalosporins or fluoroquinolones are appropriate. Anaerobic infections should be treated with metronidazole or clindamycin [4,83]. Appropriate antibiotics should be administered via IV for 2–4 weeks or a significant decrease in CRP levels, followed by oral antibiotics for a total duration of 6–12 weeks [3]. The optimal duration and route of antibiotic treatment remain debated due to a presumed association between treatment duration and relapse or failure. Further, conservative treatment should include bed rest and/or the use of an orthosis for at least 6 weeks, based on pain levels during mobilization [93]. Conservative treatments are frequently effective, but they are not always sufficient in every case.

Recurrence and mortality are key indicators of treatment success in patients with infective spondylitis [94,95]. A clinical outcome assessment needs to consider various risk factors [96]. Previous studies have revealed that medical comorbidities, undrained abscesses, and causative pathogens, such as MRSA, are significant predictors of postoperative recurrence and mortality [95,97,98]. Recurrence and mortality in older patients with infective spondylitis are significantly associated with neurological deficits; however, the severity of comorbid conditions also plays a crucial role [6,68,92,95,99,100,101].

Surgical treatment is considered in cases of acute or progressive neurological deficit, when an intraspinal lesion limits the effectiveness of conservative treatment, in instability instances, and when conservative treatment has failed. First, simple decompression or excision can be performed for an epidural abscess without spondylodiscitis, and surgical open drainage is an option. Second, only decompression or excision may be performed for an epidural abscess with spondylodiscitis in the absence of associated instability. However, posterior instrumentation is required in the presence of instability, and according to the extent of discitis, interbody fusion may be necessary. Third, a corpectomy may be performed for spondylodiscitis accompanied by bony destruction, and instrumentation of the anterior column or simultaneous instrumentation of both the anterior and posterior columns may be required [102,103].

Structural reinforcement using autobone or allobone is essential in the presence of bony destruction. The evolution of bone grafting in spinal surgeries has seen the development of various techniques and materials aimed at improving outcomes. Tricortical iliac autograft, which is a traditional bone grafting method, is widely recognized for its safety and consistently excellent results, making it the gold standard for promoting bony fusion [104]. Alternatively, structural bone allograft provides benefits such as reduced operative time and donor site morbidity elimination [105]. Recent studies indicate that combining structural bone grafts with recombinant human bone morphogenetic protein-2 (rhBMP-2) improves fusion rates [106]. However, rhBMP-2 carries potential risks, including seroma formation, radiculitis, and undesirable cell growth. Therefore, patients with a history of cancer and cerebrospinal fluid leaks or those undergoing cervical or thoracic spine surgeries should be specifically informed about these risks.

The use of transforaminal interbody debridement and fusion with antibiotic-impregnated bone graft for the treatment of pyogenic discitis and vertebral osteomyelitis has also been proposed by some clinicians, particularly in studies involving Asian populations [107].

Some researchers have proposed transpedicular curettage and drainage as an effective treatment option for infectious spondylodiscitis, particularly in patients who cannot tolerate conventional combined anterior and posterior surgeries due to multiple comorbidities, extensive infectious lesions, and poor general condition [108,109].

Vacuum-assisted closure is a form of negative pressure wound therapy with or without continuous irrigation that has appeared as a promising alternative for managing complex postoperative spinal infections. Further, it is considered a safe and effective treatment option for deep SSIs even in cases with exposed dura, especially after posterior instrumented spinal surgery [110,111,112,113,114,115,116,117,118,119].

Several strategies have been proposed to prevent postoperative infections after fusion surgery, including the application of vancomycin powder and taurolidine irrigation [120,121]; however, postoperative spine infection is more difficult to treat and exhibits a poorer prognosis than simple spine infection. The principles of open surgical debridement involve investigating the wound to identify whether the infection is deep or superficial, followed by a thorough necrotic and infected tissue removal. For early postoperative infections (within 3 months), the spinal hardware is advised not to be removed to avoid destabilizing the spine. Loose bone grafts should be removed during debridement, but graft material firmly attached to bony structures should be left in place [122]. Hardware removal for late postoperative infections is often required for several reasons. One reason is that the spinal anchorage points and the area directly beneath the rods are hard to access without hardware removal. Removing the hardware enables more thorough wound debridement if a solid fusion has occurred. Late-onset infections are frequently indolent and caused by organisms, such as coagulase-negative *Staphylococci* or *P. acnes*, which are prone to biofilm formation. Di Silvestre et al. demonstrated that not removing implants in delayed infections leaves a 50% chance of the infection to persist [123]. The advantage of removing biofilm must be weighed against the risk of destabilizing the spine before bone fusion. The hardware can be removed if fusion has occurred; however, long fusions involve a risk of fracturing the fusion mass, losing alignment, or settling into kyphosis [124]. If fusion has not occurred, an autograft and/or an allograft can be utilized to achieve bone fusion. Using an allograft does not significantly increase the postoperative infection rate [125]. Choi et al. revealed that using an intraoperative toothbrushing technique during the initial surgical debridement for postoperative spine infections can significantly lower the failure rate and decrease the necessity for revision surgery by effectively eliminating the biofilm of pathogenic bacteria [64].

In addition to multiple debridements, continuing antibiotic therapy is equally crucial. As previously mentioned, patients in stable conditions should not be given antibiotics before obtaining the culture results. However, empirical antibiotic administration is necessary for patients with sepsis and unstable conditions to prevent further clinical deterioration. Broad-spectrum antibiotics should be initiated before the final culture results are available. The duration of antibiotic therapy is debated and varies according to the type of infection. A shorter course of antibiotics is usually sufficient for postoperative infections without hardware. Postoperative discitis or osteomyelitis typically requires over 3 months of antibiotics, based on the inflammatory markers. Patients generally need at least 4–6 weeks of IV antibiotics, tailored to culture results and inflammatory markers for deep infections with hardware in place [126]. If ESR/CRP levels increase after discontinuing antibiotics and fusion has occurred, then hardware removal is recommended. Clear consensus on the duration of antibiotic treatment remains unavailable; however, a course of oral suppressive antibiotics is recommended to follow long-term IV antibiotics if hardware is retained. A shorter treatment course may be appropriate if the instrumentation is removed.

However, antibiotic-induced allergic reactions can increase inflammatory markers, and the use of broad-spectrum antibiotics may cause antibiotic-related diarrhea due to *Clostridioides difficile*; thus, these potential issues should be considered [127,128,129,130,131,132]. Hensgens et al. reported that antibiotic administration increases the risk for *C. difficile* infection not only while taking them but also for up to three months after stopping, with the highest risk occurring during treatment and in the first month afterward [133]. Also, the use of multiple antibiotics has been associated with an increased risk of Clostridium difficile infection [134].

In patients requiring surgical treatment for infective spondylitis after previous spinal instrumented surgery, a decision must be made between a less invasive noninstrumented approach, which retains the previous instrumentation, or a more invasive additional instrumented surgery which involves thorough infected tissue removal and stabilization. Spinal infections frequently occur in elderly patients with comorbidities, making clinical decision-making challenging because the use of instrumentation can inadvertently interfere with infection management by promoting biofilm formation [67,135]. Outcomes vary according to factors, such as the presence of severe comorbidities, psoas abscess, and MRSA infection; however, studies have revealed comparable recurrence and mortality rates between patients who undergo additional instrumentation and those in the noninstrumented surgery group. Therefore, individual risk factors need to be carefully considered when selecting the most appropriate surgical approach for each patient [68].

Surgical intervention is rarely required in cases of isolated psoas abscesses. Instead of immediate surgery, nonsurgical treatments, such as antibiotics and percutaneous drainage, are prioritized. However, caution is required as infective spondylitis may be the primary source of infection [136,137,138].

Hyperbaric oxygen therapy has been applied as a supplementary treatment for spinal infections. This therapy may help in infection treatment by restoring intramedullary bone oxygen tension, normalizing phagocyte activity, stimulating neovascularization at the edges of healing wounds, inducing vasodilation in healing tissues, and suppressing biofilm formation. Side effects associated with this adjunct therapy were not reported [139]. Further research is warranted to determine if hyperbaric oxygen therapy reduces the number of revision surgeries or debridements and improves long-term outcomes compared with standard treatment alone.

## 10. Conclusions

Universally applicable guidelines for managing spinal infections remain unavailable because of the diverse and often comorbid nature of the patient population and the broad range of treatment options available, making treatment challenging. Patients should consider both medical and surgical options based on a review of the literature and personal experience. In cases of neurological symptom worsening or medical treatment failure, surgery may be required with or without instrumentation. Patients who have severe comorbidities limit invasive procedures; thus, a less invasive approach, such as transpedicular irrigation, can be a viable alternative. More prospective randomized trials are warranted to confirm and refine these treatment strategies.

## Data Availability

Not applicable.
